# Time-series analysis of the impact of short-term exposure to atmospheric pollutants on stroke risk among residents in Guiyang City, 2016–2021

**DOI:** 10.3389/fpubh.2026.1777772

**Published:** 2026-10-16

**Authors:** Ling Zhang, Minmin Su, Shuangfei Zhai, Meiqin Jiang, Xuanhao Chen, Ruifang Liu, Gang Chen, Jianhua Zhang

**Affiliations:** 1 School of Basic Medicine, Guizhou University of Traditional Chinese Medicine, Guiyang, Guizhou, China; 2 Guizhou Center for Disease Control and Prevention, Guiyang, Guizhou, China; 3 Guiyang Municipal Center for Disease Control and Prevention, Guiyang, Guizhou, China

**Keywords:** air pollution, generalized additive model, incidence risk, lag effect, stroke, time-series analysis

## Abstract

**Background:**

Stroke is one of the leading causes of death among Chinese residents, and air pollution is a significant environmental risk factor. Currently, research on the association between air pollution and stroke incidence remains limited in low-pollution karst cities in Southwest China. This study aims to evaluate the short-term effects of major air pollutants on stroke incidence in Guiyang City.

**Methods:**

Daily data on stroke incidence, air pollutants, and meteorological conditions for permanent residents of Guiyang from 2016 to 2021 were collected. A time-series analysis was conducted using a generalized additive model (GAM) based on a quasi-Poisson distribution. Long-term trends, meteorological factors, day-of-week effects, and holiday effects were controlled. The relative risk (*RR*) and 95% *CI* for stroke incidence per 10 μg/m^3^ increase in pollutant concentration (CO: 1 mg/m^3^) were assessed. Single-day and cumulative lag models were used to examine lag effects, and analyses were stratified by gender and age.

**Results:**

A total of 11,517 stroke cases were reported, with a daily average of 5.25 cases. Short-term exposure to PM₂.₅, PM₁₀, SO₂, NO₂, and O₃ (per 10 μg/m^3^ increase) and CO (per 1 mg/m^3^ increase) was positively associated with stroke risk. The *RRs* (95% *CI*) at maximum lag were: PM₂.₅ 1.026 (1.005–1.048), PM₁₀ 1.015 (1.001–1.030), SO₂ 1.075 (1.016–1.137), NO₂ 1.057 (1.017–1.098), O₃ 1.013 (1.003–1.023), and CO 1.346 (1.072–1.691). Women were more sensitive to all six pollutants, especially CO (lag0 *RR* = 1.548, 95% *CI*: 1.116–2.147). Older adults (≥65 years) showed increased risk across all pollutants, while only NO₂ and PM₁₀ had statistically significant effects on those <65 years.

**Conclusion:**

Even under low pollution levels, short-term exposure to air pollutants is significantly associated with increased stroke risk with a lag effect, particularly among women and older adults. Strengthening air quality management and protection for high-risk populations is recommended to potentially reduce the burden of stroke.

## 1 Introduction

Stroke, also known as cerebrovascular accident, is an acute cardiovascular disease characterized by sudden rupture of cerebral blood vessels or obstruction of blood flow to the brain, resulting in brain tissue damage. Based on pathological nature, it is typically classified into ischemic stroke (cerebral infarction) and hemorrhagic stroke (1). Data from the 2019 Global Burden of Disease (GBD) study indicates that stroke ranks as the second leading cause of death worldwide and is the primary cause of both mortality and disability in China. Characterized by high incidence, mortality, recurrence, and disability rates, stroke severely impacts patients’ physical health and quality of life, making it a significant public health issue in China (2, 3). Reports indicate approximately 2.5 million new stroke cases occur annually in China, with 1.6 million deaths attributable to stroke (4). Stroke pathogenesis involves not only traditionally recognized intrinsic factors such as hypertension, heart disease, diabetes, obesity, and unhealthy lifestyles (5), but also environmental hazards. Air pollution has been identified as a significant environmental factor associated with acute stroke onset (6). Although numerous studies worldwide have explored the association between air pollution and stroke, most have used mortality or hospitalization rates as outcomes, with relatively few studies based on incidence data (7). Furthermore, existing research has primarily focused on heavily polluted or economically developed cities in China, with limited studies conducted in cities with relatively lower air pollution levels (8–11).

Guiyang features typical karst topography, characterized by the coexistence of mountains and valley basins. This type of landform may lead to temperature inversions and reduced atmospheric ventilation, thereby limiting the dispersion of air pollutants. Compared with plain cities, this unique geographical environment may alter the health effects of air pollution (12–14). However, there is currently insufficient evidence to determine whether the short-term effects of air pollutants on stroke incidence persist in cities like Guiyang, which exhibit overall low pollution levels alongside unique geographical, climatic, and socioeconomic characteristics. Therefore, against this backdrop, this study utilized air pollutant and stroke incidence data from Guiyang City between 2016 and 2021. For the first time in a low-pollution karst topography city, it systematically employed a Poisson-based generalized additive model (GAM) to assess the short-term association between air pollutants and stroke risk among Guiyang residents. Additionally, this study assessed potential effects through stratified analyses by gender and age to identify susceptible populations. This research not only provides crucial empirical evidence on the health effects of air pollution in cities with low pollution levels but also offers a scientific basis for developing targeted public health intervention strategies under specific geographical and climatic conditions.

## 2 Materials and methods

### 2.1 Study area

The research site for this study is located in Guiyang City. Situated in the eastern part of the Yunnan-Guizhou Plateau in Southwest China, Guiyang lies between 106°07′ and 107°17′ east longitude and 26°11′ and 27°22′ north latitude. As the capital of Guizhou Province, Guiyang serves as the province’s political, economic, cultural, educational, scientific, technological, and transportation hub. It also functions as a major transportation and communications hub, industrial base, and commercial and tourism service center in Southwest China. The city administers six main urban districts: Huaxi District, Wudang District, Baiyun District, Nanming District, Yunyan District, and Guanshanhu District. With a total area of 8,043.45 square kilometers, Guiyang had a permanent resident population of 6.1023 million at the end of 2021 and an average annual population of 6.0461 million. The climate is characterized as subtropical, humid and temperate, with additional features of a plateau monsoon climate. The average annual precipitation is 1,173.9 mm, the annual mean temperature is 15.16 °C, and the average annual humidity is 80%. The terrain slopes from higher elevations in the southwest to lower elevations in the northeast, with an average altitude of 1,000 meters in the study area. The region exhibits well-developed karst topography. These terrain and climatic conditions limit the dispersion of environmental air pollutants.

### 2.2 Data sources

#### 2.2.1 Stroke incidence monitoring data

Daily incidence data for cerebrovascular accidents among the permanent resident population of Guiyang City from January 1, 2016, to December 31, 2021, are sourced from the China Chronic Disease and Related Risk Factor Surveillance System. This data was generated by integrating case reports submitted by medical institutions at all levels in Guiyang City (including emergency departments, outpatient clinics, and inpatient wards). It includes detailed information such as gender, age, current/permanent address, disease diagnosis type, ICD diagnosis codes, and date of onset. Following the coding rules of the International Statistical Classification of Diseases and Related Health Problems, 10th Revision (ICD-10), patients with ICD codes I60–I64 (cerebrovascular disease) were included as study subjects. Subgroup analyses were conducted based on gender and age. Following World Health Organization (WHO) standards, the older adult group was defined as ≥65 years old, while the non-older adult group was defined as <65 years old. During the study period, both the stroke incidence data and the air pollutant data were complete, with no missing values. This data segment underwent encryption processing to strictly safeguard personal privacy and does not involve ethical approval and informed consent.

#### 2.2.2 Air pollution and meteorological monitoring data

This study collected environmental air pollutant data for Guiyang City from January 1, 2016, to December 31, 2021. The environmental air pollution data were sourced from the Guiyang Municipal Environmental Protection Bureau. Twelve environmental monitoring stations were established across the urban area: Tongmuling, Biyunwo, Zhongyuan Village, Xinhua Road, Municipal Environmental Protection Station, Taici Bridge, Ma’anshan, Hongbianmen, Yanzi Chong, Jianhu Road, Shapo Station, and Yunfeng Station. In this study, the daily average concentrations of air pollutants—sulfur dioxide (SO₂), nitrogen dioxide (NO₂), fine particulate matter (PM₂.₅), inhalable particulate matter (PM₁₀), carbon monoxide (CO), and ozone (O₃) are the average daily concentrations across all 12 monitoring stations. Except for O₃, which uses an 8-h average, all other pollutants employ 24-h average concentrations calculated from all valid monitoring stations. CO is measured in mg/m^3^, while other air pollutants are measured in μg/m^3^. Concurrent meteorological data, including daily average temperature (°C), daily average atmospheric pressure (hPa), daily average relative humidity (%) and daily average wind speed (m/s), were obtained from the Guiyang Meteorological Bureau.

### 2.3 Statistical methods

#### 2.3.1 Descriptive statistical analysis

Statistical descriptions of air pollutant data, meteorological data, total incidence population, and incidence data by gender and age group were performed using mean ± standard deviation ( $\bar{x}$ **± s**), minimum (Min), maximum (Max), and percentiles (*P_25_*, *P_50_*, *P_75_*). Time-series plots of air pollutants and meteorological factors were also generated. To examine correlations between air pollutants and meteorological factors, Spearman’s correlation coefficient was used to assess relationships between pollutants and meteorological data. A two-tailed test was applied, with *p* < 0.05 indicating statistically significant differences.

#### 2.3.2 Statistical analysis

No imputation methods were used in this study. As both the stroke incidence data and the air pollutant data were complete with no missing values, all analyses were based on the original observed data. Given that the daily incidence of stroke among residents is a low-probability event relative to the permanent population of Guiyang and remains relatively stable, we did not include a population offset term in this study. Instead, a smooth function of time (ns (*t*, df = 7/year)) was employed to control for long-term trends, and the actual distribution approximates a Poisson distribution. Therefore, this study utilized a time-series generalized additive model (GAM) based on a quasi-Poisson distribution to investigate the association between short-term exposure to air pollutants (PM₂.₅, PM₁₀, CO, O₃, SO₂, NO₂) and the risk of stroke incidence. The model incorporates smoothing functions to account for nonlinear effects while controlling for confounders such as average temperature, average relative humidity, long-term trends, public holiday effects, and day-of-week effects, thereby minimizing potential interference with the analysis results. The structure of the GAM established in this study is as follows:

$$\begin{array}{c} \log[E(Y_{t})]=\alpha +\beta^{*}\text{Pollutan}t_{t}+\mathrm{ns}(\text{Temperature},3)+\\ \mathrm{ns}(\text{Humidity},3)+\mathrm{ns}(\text{time},6^{*}7)+\\ \mathrm{as}.\text{factor}(\text{Week})+\mathrm{as}.\text{factor}(\text{Holiday}) \end{array}$$

In the above equation: log() denotes the link function, specifically the quasi-Poisson distribution; *E*(*Y_t_*) represents the expected number of stroke cases on day *t*; *t* indicates the observation date; *Y_t_* is the actual number of stroke cases on day *t*; *α* is the model intercept; *β* is the regression coefficient for the pollutant term; *Pollutiont_t_* denotes the concentration of a specific air pollutant on day *t*; *ns*() represents the natural cubic spline function, controlling for the effects of nonlinear confounding factors such as long-term temporal trends, temperature, and humidity on stroke incidence; *df* denotes degrees of freedom; time reflects the long-term temporal trend; *Temperature* represents the mean temperature variable; *Humidity* denotes the mean relative humidity variable; *Week* accounts for the day-of-week effect; *Holiday* accounts for the public holiday effect. Based on prior research findings and incorporating the Akaike Information Criterion (AIC) to determine the degrees of freedom for time trends and meteorological factors (mean temperature, mean relative humidity) (15, 16), this study The degrees of freedom for time trend (7 per year), temperature (3), and humidity (3) were selected based on the Akaike Information Criterion (AIC) using Poisson family models, as quasi-Poisson does not yield AIC. The combination with the lowest AIC was chosen see Supplementary Table S1 for detailed AIC comparisons. Overdispersion was assessed by examining the dispersion parameter in each quasi-Poisson model see Supplementary Table S2; values >1 indicate overdispersion and justify the use of quasi-Poisson. Residual autocorrelation was examined using partial autocorrelation function (PACF) plots (Supplementary Figures S1). Model diagnostics, including residual plots and partial residual plots, were also examined to verify model assumptions see Supplementary Figures S2–S4. Dummy variables were employed to control for the effects of day of the week and public holidays.

Due to the persistent and delayed nature of health effects from ambient air pollutants, their impacts may gradually manifest over several days or even weeks following exposure (17, 18). Based on existing research, a maximum lag duration of 7 days was selected (18). The relative risk (*RR*) and 95% confidence interval (95% *CI*) were calculated for single-day lag effects (lag0–lag7) and cumulative lag effects (lag01–lag07). The lag time at which the strongest association between air pollutants and stroke incidence was observed among residents was determined according to the principle of the highest *RR* value. Additionally, we conducted stratified analyses to investigate the effects of air pollution on different genders (male and female) and age groups (≥65 years and <65 years). Additionally, to address potential confounding due to high correlations among pollutants (e.g., PM₂.₅ and PM₁₀, *r* = 0.948), this study conducted two-pollutant model fitting based on the single-day lag model (Lag0) for the primary outcome (total stroke events). Finally to account for multiple comparisons due to testing six pollutants and stratified analyses, we applied the Benjamini-Hochberg false discovery rate (FDR) correction to the raw *p*-values from the main analysis (total population, lag0). Associations with an FDR-adjusted *p*-value < 0.05 were considered statistically significant.

This study employed R version 4.4.2 for statistical analysis, utilizing the “mgcv” package to construct generalized additive models and the “splines” package to implement natural cubic spline functions. Graphical representations were generated using the ggplot2 package. A two-tailed test with a significance level of *α* = 0.05 was applied; differences were considered statistically significant if *p* < 0.05.

#### 2.3.3 Sensitivity analysis

To validate the robustness of the research findings, this study conducted sensitivity analyses by adjusting the degrees of freedom for time trends (6–8 times per year), temperature (3–6 times), and humidity (3–6 times) within the single-pollutant model to assess the model’s robustness. Additionally to assess the potential impact of the COVID-19 pandemic, we additionally conducted a period-specific sensitivity analysis by stratifying the study period into pre-pandemic (2016–2019) and pandemic (2020–2021) phases, and compared the effect estimates with the full-period results.

## 3 Results

### 3.1 Basic overview of stroke incidence in Guiyang City, 2016–2021

From 2016 to 2021, Guiyang City reported a total of 11,517 stroke cases, with an average daily incidence of 5.25 cases. Among these, 6,657 cases (57.80%) occurred in males and 4,860 cases (42.20%) in females. 7,325 cases (63.60%) occurred in individuals aged ≥65 years, while 4,192 cases (36.40%) occurred in those <65 years. The overall incidence of stroke showed an upward trend; for details, see Supplementary Figures S5. The basic characteristics of stroke incidence are presented in Table 1.

**Table 1:** Basic data on stroke incidence in Guiyang City, 2016–2021.

Variable	*N* (%)	$\bar{x}$ ± s	Min	*P_25_*	*P_50_*	*P_75_*	Max
Daily number of stroke cases							
Total	11,517 (100)	5.25 ± 6.60	0.00	2.00	3.00	6.00	54.00
Gender							
Male	6,657 (57.80)	3.04 ± 4.06	0.00	1.00	2.00	3.00	35.00
Female	4,860 (42.20)	2.22 ± 2.96	0.00	0.00	1.00	3.00	24.00
Age							
≥65 years old	7,325 (63.60)	3.34 ± 4.32	0.00	1.00	2.00	4.00	34.00
<65 years old	4,192 (36.40)	1.91 ± 2.68	0.00	0.00	1.00	2.00	23.00

### 3.2 Overview of atmospheric pollutants and meteorological factors in Guiyang City, 2016–2021

The daily average concentrations of PM_2.5_, PM_10_, SO₂, NO₂, O₃, and CO in Guiyang City from 2016 to 2021 were 29.29 μg/m^3^, 50.12 μg/m^3^, 11.29 μg/m^3^, 21.92 μg/m^3^, 75.81 μg/m^3^, and 0.63 mg/m^3^. According to the Environmental Air Quality Standards (GB 3095–2012), the annual average concentrations of PM _2.5_, PM_10_, SO₂, NO₂, O₃, and CO in Guiyang during 2016–2021 did not exceed the national Grade II concentration limits. The daily average temperature was 15.16 °C, the average relative humidity was 80.38%, and the average atmospheric pressure was 877.81 hPa, as shown in Table 2. During the study period, all air pollutants except O₃ exhibited higher concentrations in winter and spring and lower concentrations in summer and autumn. O₃, however, showed higher concentrations in summer and autumn and lower concentrations in winter and spring. Atmospheric pressure trends were opposite to those of temperature and humidity (Supplementary Figures S6, S7).

**Table 2:** Descriptive statistics of air pollutants and meteorological factors in Guiyang City, 2016–2021.

Variables	$\bar{x}$ ± s	Min	*P_25_*	*P_50_*	*P_75_*	Max
Air pollutants						
PM_2.5_ (μg/m^3^)	29.29 ± 16.67	5.00	16.73	26.09	37.74	152.80
PM_10_ (μg/m^3^)	50.12 ± 25.46	10.08	30.92	44.75	63.25	203.83
SO_2_ (μg/m^3^)	11.29 ± 8.35	3.58	6.00	8.25	13.36	87.92
NO_2_ (μg/m^3^)	21.92 ± 8.57	4.75	15.82	20.17	26.36	66.42
O_3_ (μg/m^3^)	75.81 ± 30.68	9.90	53.75	71.20	95.83	182.00
CO (mg/m^3^)	0.63 ± 0.16	0.31	0.52	0.60	0.71	1.57
Meteorological factors						
Temperature (°C)	15.16 ± 7.45	−3.70	9.00	15.90	22.00	28.20
Air pressure (hPa)	877.81 ± 5.15	865.10	873.60	877.50	881.60	895.20
Relative humidity (%)	80.38 ± 11.85	39.20	72.80	81.00	89.68	100.00

### 3.3 Spearman correlation between atmospheric pollutants and meteorological factors

Spearman correlation analysis revealed statistically significant correlations (*p* < 0.01) among daily concentrations of the six pollutants. Except for O₃, all pollutants showed significant positive correlations (*p* < 0.01), with PM₂.₅ exhibiting the strongest correlation with PM₁₀ (*r* = 0.948, *p* < 0.01). Average temperature and average relative humidity showed negative correlations with most pollutants. However, O₃ exhibited a positive correlation with average temperature (*r* = 0.394), and CO showed a positive correlation with average relative humidity (*r* = 0.224). Average temperature correlated negatively with both average air pressure and average relative humidity. Average air pressure correlated positively with average relative humidity (*r* = 0.091). Detailed results are presented in Table 3 and Supplementary Figures S8.

**Table 3:** Spearman correlation analysis between air pollutants and meteorological factors.

Variables	PM_2.5_	PM_10_	SO_2_	CO	NO_2_	O_3_	Air Pressure	Temperature	Relative humidity
PM_2.5_	1	0.948**	0.680**	0.562**	0.667**	0.361**	0.301**	−0.358**	−0.342**
PM_10_		1	0.593**	0.423**	0.690**	0.507**	0.202**	−0.176**	−0.528**
SO_2_			1	0.628**	0.566**	−0.017**	0.558**	−0.703**	−0.135**
CO				1	0.564**	−0.152**	0.236**	−0.502**	0.224**
NO_2_					1	0.170**	0.207**	−0.237**	−0.187**
O_3_						1	−0.220**	0.394**	−0.726**
Air pressure							1	−0.769**	0.091**
Temperature								1	−0.354**
Relative humidity									1

** indicates *p* < 0.01 (two-tailed).

### 3.4 Lag effects of air pollutants exposure on stroke risk among residents

The association between short-term exposure to six air pollutants and the risk of stroke onset among residents is shown in Figure 1. Complete results of different lag models are shown in Supplementary Table S3. Among single-day lagged effects, the maximum effect lag times for Guiyang’s PM₂.₅, PM₁₀, SO₂, NO₂, CO, and O₃ were lag0, lag0, lag0, lag0, lag0, and lag1, respectively. Their *RR* and 95% *CI* were 1.026 (1.005–1.048), 1.015 (1.001–1.030), 1.075 (1.016–1.137), 1.057 (1.017–1.098), 1.346 (1.072–1.691), and 1.013 (1.003–1.023), respectively. Among these pollutants, CO showed the strongest association, followed by SO₂. In the multi-day cumulative lag effects, PM₂.₅, PM₁₀, SO₂, and NO₂ exhibited the strongest effects at lags ranging from 0 to 4 days (Lag04), with corresponding *RR* values and 95% *CIs* of 1.033 (1.004–1.062), 1.019 (1.001–1.037), 1.098 (1.014–1.189), and 1.076 (1.019–1.135), respectively. For O₃, the lagged effect at 0 to 1 day (Lag01) showed the strongest impact on the entire population, with an *RR* of 1.015 (95% *CI*: 1.002–1.028), *p* < 0.05. After applying the FDR correction for multiple testing, PM₂.₅, PM₁₀, SO₂, NO₂, and CO remained statistically significant (FDR < 0.05), while O₃ did not see Supplementary Table S4. This indicates that the associations for the major pollutants are robust to concerns regarding multiple testing.

**Figure 1:**
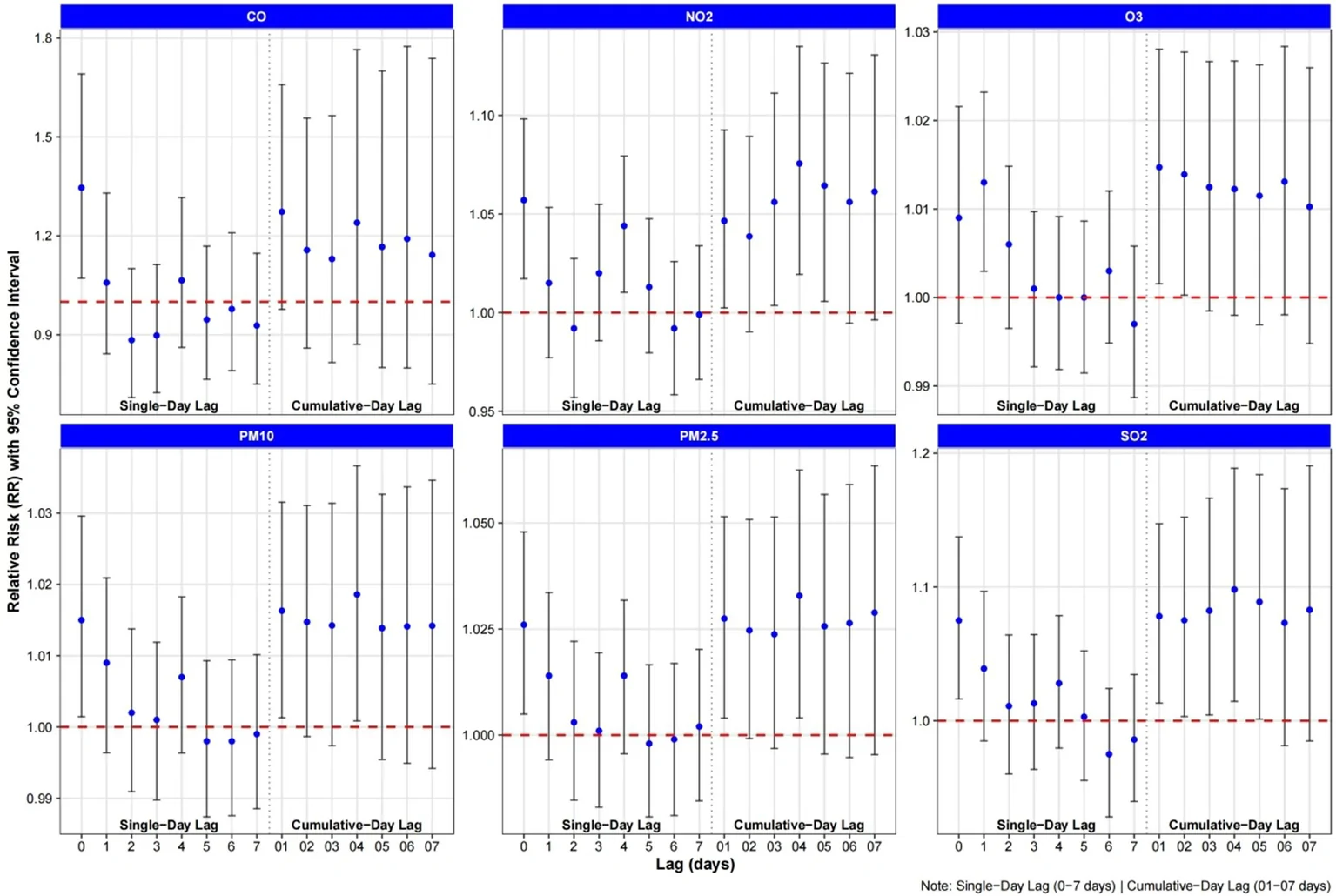
Lag effect diagram of the association between atmospheric pollutants and stroke risk in the general population of Guiyang City, 2016–2021.

### 3.5 Results of two-pollutant model analyses

To further explore the stability of the effects of major pollutants and address potential multicollinearity, we conducted two-pollutant model analyses at Lag0, the lag day showing the strongest association for PM₂.₅, NO₂, and CO. The results are presented in Table 4. After mutual adjustment, the effects of PM₂.₅ and NO₂ were attenuated and lost statistical significance. The *RR* for PM₂.₅ decreased from 1.026 (95% *C*I: 1.005–1.048) in the single-pollutant model to 1.011 (95% *CI*: 0.984–1.039) after adjusting for NO₂ and CO. Similarly, the *RR* for NO₂ decreased from 1.057 (95% *CI*: 1.017–1.098) to 1.042 (95% *CI*: 0.994–1.093) after adjusting for PM₂.₅. Notably, the effect of CO was substantially attenuated after adjustment for other factors. The *RR* for CO dropped from 1.346 (95% *CI*: 1.072–1.691) in the single-pollutant model to 1.068 (95% *CI*: 0.784–1.456) in the two-pollutant model adjusted for PM₂.₅ and NO₂, representing a reduction of 20.63% in the effect estimate. Despite the attenuation, variance inflation factors (VIFs) for all two-pollutant models were below 3 (PM₂.₅ VIF = 2.06, NO₂ VIF = 2.07, CO VIF = 1.66), indicating that the observed attenuation was not primarily driven by statistical multicollinearity.

**Table 4:** Comparison of effect estimates from single-pollutant and multi-pollutant models for stroke risk at lag0 (total population).

Pollutant	Single-pollutant *RR* (95% *CI*)	Multi-pollutant *RR* (95% *CI*)	Adjustment	RR change (%)	VIF (PM₂.₅)	VIF (NO₂)	VIF (CO)
PM₂.₅	1.026 (1.005–1.048)	1.011 (0.984–1.039)	NO₂ + CO	−1.45	2.06	2.07	1.66
NO₂	1.057 (1.017–1.098)	1.042 (0.994–1.093)	PM₂.₅	−1.37	2.06	2.07	1.66
CO	1.346 (1.072–1.691)	1.068 (0.784–1.456)	PM₂.₅ + NO₂	−20.63	2.06	2.07	1.66

VIF < 5 indicates that multicollinearity is weak.

### 3.6 Stratified analysis

#### 3.6.1 Gender stratification analysis

The results of the gender-stratified analysis are shown in Figure 2 and Supplementary Table S5. Neither PM_2.5_, PM_10_, SO₂, nor O₃ had a significant effect on males. Only CO and NO₂ affected males, with CO showing significance on day 4 after exposure (lag4). The corresponding *RR* values were 1.327 (95% *CI*: 1.026–1.717); NO₂ showed significant effects on males at lag 4 and cumulative lag 0 to 7 days (lag07), with corresponding *RR* values and 95% *CIs* of 1.056 (95% *CI*: 1.015–1.100) and 1.090 (1.009–1.178). Notably, NO₂ exerted its greatest impact on stroke incidence among males on lag day 7 (lag07). The maximum lag periods for PM_2.5_, PM_10_, SO₂, NO₂, CO, and O₃ effects on females were Lag04, Lag01, Lag04, Lag03, Lag0, and Lag01, respectively. The corresponding *RR* values and 95% *CIs* were 1.050 (1.008–1.094), 1.024 (1.002–1.064), 1.171 (1.046–1.310), 1.076 (1.000–1.158), 1.548 (1.116–2.147), and 1.026 (1.007–1.046), respectively. Among these, CO was the pollutant most strongly associated with stroke incidence in both men and women. All six pollutants showed statistically significant effects on female residents (*p* < 0.05).

**Figure 2:**
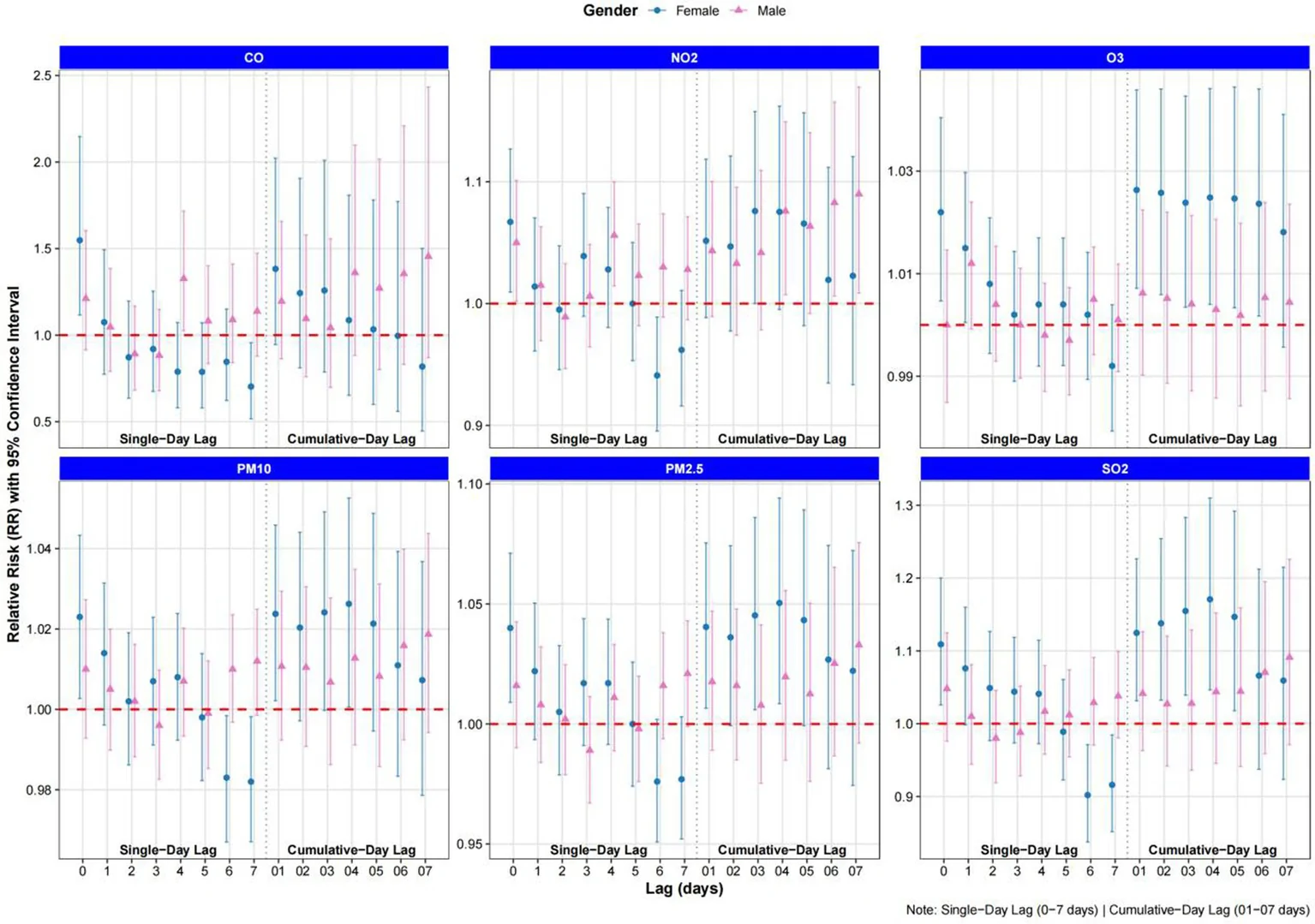
Effects of short-term air pollution exposure on stroke risk among residents of different genders in Guiyang City [*RR* (95% *CI*)].

#### 3.6.2 Age stratification analysis

The age-stratified results are shown in Figure 3 and Supplementary Table S6. For the ≥65 age group, the maximum effect lag times for PM_2.5_, PM_10_, SO₂, NO₂, CO, and O₃ were Lag0, Lag0, Lag0, Lag0, Lag0, and Lag01, respectively. The corresponding *RR* values and their 95% *CIs* were 1.029 (1.003–1.055), 1.016 (1.000–1.033), 1.130 (1.060–1.205), 1.059 (1.012–1.108), 1.312 (1.001–1.720), and 1.013 (1.001–1.025), respectively. No statistically significant association was found between air pollutants PM_10_, SO₂, CO, or O₃ and stroke incidence in individuals aged <65 years. Only NO₂ and PM_10_ showed short-term effects: PM_10_ exerted influence at lag 0 to 4 days (Lag04) with an *RR* of 1.027 (95% *CI*: 1.000–1.055). NO₂ showed effects at Lag0 (same day) and Lag01 (lag 0–1 days), with *RR* values of 1.070 (95% *CI*: 1.018–1.124) and 1.013 (95% *CI*: 1.018–1.196), respectively (*p* < 0.05).

**Figure 3:**
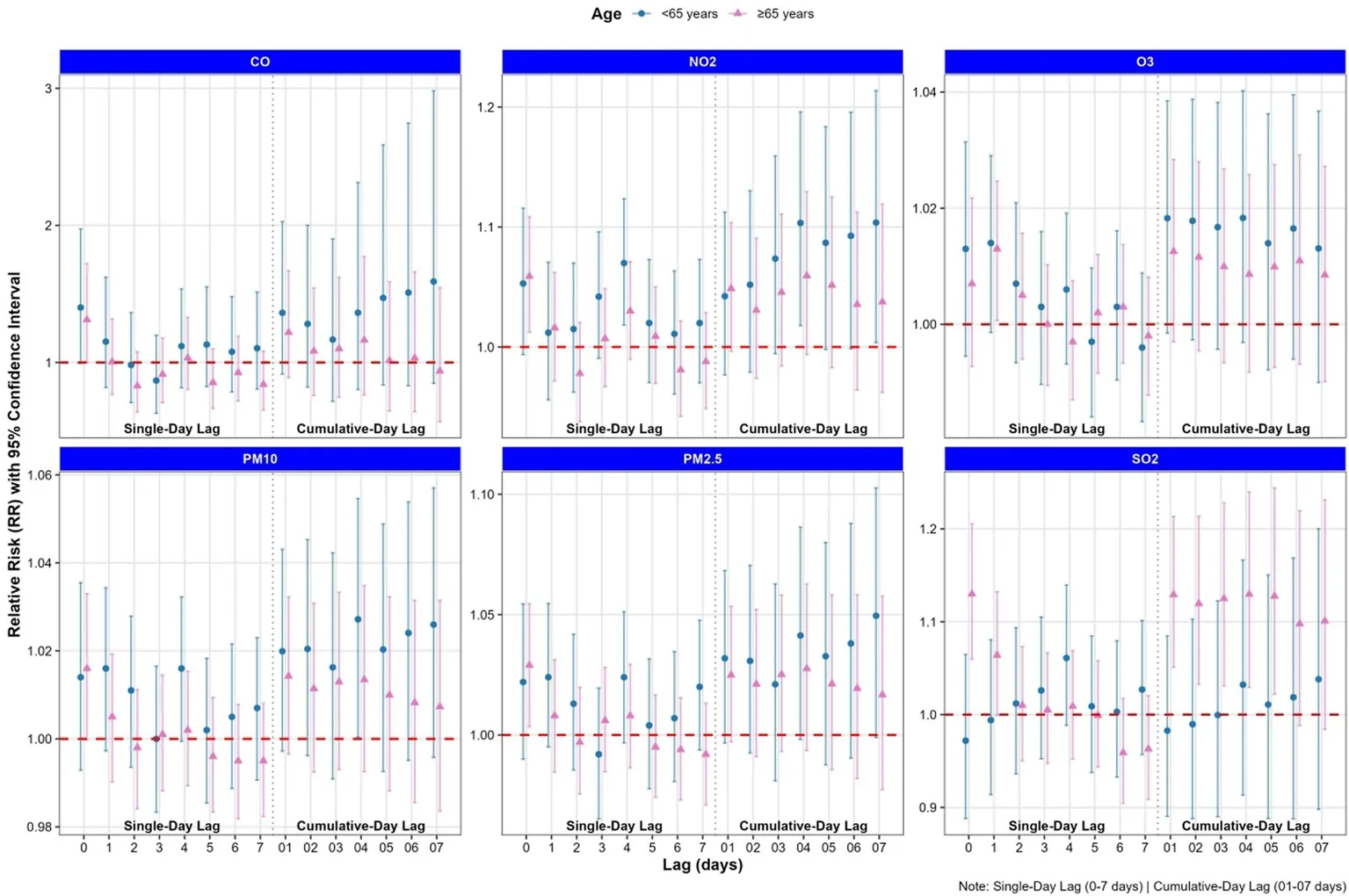
Effects of short-term air pollution exposure on stroke risk among residents of different ages in Guiyang City [*RR* (95% *CI*)].

### 3.7 Sensitivity analysis

Sensitivity analysis results indicate that by altering the degrees of freedom for time (df = 6–8), temperature (df = 3–5), and humidity (df = 3–5), minimal changes were observed in the relative risk (*RR*) values for primary pollutants (PM₂.₅, PM₁₀, SO₂, NO₂, CO, and O₃) showed minimal variation in relative risk, with only slight fluctuations in effect estimates. After adjusting for degrees of freedom, *RR* values exhibited negligible variation, indicating model robustness. See Table 5. The period-stratified analysis showed that during the pre-pandemic period (2016–2019), PM₂.₅, PM₁₀, NO₂, and SO₂ were significantly associated with increased stroke risk, with effect estimates generally consistent with those from the full study period (Supplementary Table S7). During the pandemic period (2020–2021), PM₂.₅ and NO₂ remained statistically significant, while the associations for PM₁₀, SO₂, CO, and O₃ were attenuated and no longer significant, likely due to reduced statistical power from the shorter observation period. O₃ remained non-significant across all periods. Overall, these findings suggest that the COVID-19 pandemic did not materially alter the main conclusions of this study.

**Table 5:** Sensitivity analysis of relative risk (*RR*, 95% *CI*) for air pollutants at different levels of freedom.

Variables	SO_2_	NO_2_	PM_2.5_	PM_10_	CO	O_3_
df for time						
6	1.080 (1.021–1.143)	1.063 (1.023–1.104)	1.032 (1.011–1.054)	1.019 (1.006–1.034)	1.392 (1.108–1.749)	1.013 (1.001–1.025)
7	1.074 (1.015–1.137)	1.057 (1.017–1.099)	1.027 (1.005–1.048)	1.015 (1.001–1.030)	1.311 (1.044–1.646)	1.010 (0.998–1.022)
8	1.071 (1.012–1.133)	1.054 (1.014–1.095)	1.027 (1.005–1.049)	1.016 (1.002–1.036)	1.296 (1.033–1.626)	1.013 (1.001–1.025)
df for temperature						
3	1.074 (1.015–1.137)	1.057 (1.017–1.099)	1.027 (1.005–1.048)	1.015 (1.001–1.030)	1.311 (1.044–1.646)	1.010 (0.998–1.022)
4	1.088 (1.028–1.150)	1.065 (1.025–1.107)	1.026 (1.005–1.048)	1.016 (1.002–1.030)	1.339 (1.068–1.679)	1.007 (0.995–1.019)
5	1.090 (1.031–1.153)	1.066 (1.026–1.107)	1.025 (1.004–1.047)	1.015 (1.001–1.029)	1.316 (1.049–1.651)	1.005 (0.993–1.018)
6	1.087 (1.028–1.150)	1.062 (1.022–1.104)	1.022 (1.001–1.044)	1.014 (1.000–1.028)	1.278 (1.016–1.607)	1.003 (0.991–1.016)
df for humidity						
3	1.074 (1.015–1.137)	1.057 (1.017–1.099)	1.027 (1.005–1.048)	1.015 (1.001–1.030)	1.311 (1.044–1.646)	1.010 (0.998–1.022)
4	1.074 (1.015–1.137)	1.057 (1.018–1.099)	1.027 (1.006–1.049)	1.016 (1.002–1.030)	1.314 (1.046–1.650)	1.010 (0.998–1.023)
5	1.074 (1.015–1.137)	1.057 (1.017–1.099)	1.027 (1.005–1.049)	1.015 (1.001–1.030)	1.313 (1.044–1.651)	1.010 (0.998–1.023)
6	1.074 (1.015–1.137)	1.057 (1.017–1.098)	1.026 (1.005–1.048)	1.015 (1.001–1.029)	1.308 (1.040–1.645)	1.010 (0.998–1.022)

The RR values represent the maximum effect estimates from the single-day lag models (lag0 to lag7). The concentration units for PM_2.5_, PM_10_, NO₂, SO₂, and O₃ are 10 μg/m^3^, while the concentration unit for CO is 1 mg/m^3^.

## 4 Discussion

This study comprehensively examines the relationship between six major air pollutants and stroke incidence among residents of Guiyang City for the first time. Based on stroke incidence and air pollutant monitoring data from Guiyang City between 2016 and 2021, it employs a generalized additive model (GAM) based on a Poisson distribution within a time-series research framework. To our knowledge, this is the first systematic study in Guizhou Province examining the association between short-term exposure to environmental pollution and stroke. Findings reveal significant positive correlations between short-term exposure to PM₂.₅, PM₁₀, SO₂, NO₂, CO, and O₃ and stroke incidence risk. Furthermore, subgroup analyses revealed that these associations were stronger among women and older adults. As the first relevant time-series study in a low-pollution, high-altitude karst city in southwest China, this research not only provides crucial empirical evidence on the health effects of air pollution under unique geographical and climatic conditions but also offers scientific support for developing public health strategies to prevent and control stroke in low-pollution areas.

This study is the first to reveal short-term associations between multiple air pollutants and stroke incidence in a low-pollution karst topography urban system. Results indicate that even when pollutant concentrations fall below national standards, short-term exposure is significantly associated with increased stroke risk, consistent with findings from highly polluted regions (19–21). Differences in pollutant lag effects were observed: PM₂.₅, PM₁₀, SO₂, and NO₂ exhibited the strongest cumulative effects on stroke risk during days 0 to 4 post-exposure (lag04), suggesting persistent associations between these pollutants and stroke. Conversely, O₃ exhibited the greatest effect on the entire population during lag 0 to 1 days (Lag01), with a *RR* of 1.015 (95% *CI*: 1.002–1.028), *p* < 0.05. This discrepancy may relate to differing pollutant pathways. Particulate matter and gaseous pollutants like SO₂ and NO₂ may rapidly induce vascular events by promoting hypercoagulability and triggering acute inflammation. Studies, including that of Liu et al. (4), consistently demonstrate significant effects of particulate and gaseous pollutants on stroke hospitalization risk within several days of exposure. This suggests persistent damage to the cerebrovascular system by such pollutants, potentially inducing acute stroke through cumulative long-term inflammatory responses and oxidative stress (22, 23). In contrast, O₃, as a potent oxidant, may require prolonged inflammatory cascades to damage the cardiovascular system, explaining its delayed effects—a characteristic also observed in Guo et al.’s (10) study on ischemic stroke in South China. This phenomenon likely relates to O₃'s potent oxidizing properties, as short-term exposure can rapidly disrupt vascular endothelial function, subsequently triggering thrombosis or vasospasm (24).

CO showed the strongest association at lag 0 days (*RR* = 1.346, 95% *CI*: 1.072–1.691), representing the factor most strongly associated with risk factor among all pollutants. This finding aligns with the mechanistic study by Kamat et al. (25): CO directly damages cerebral vascular homeostasis through pathways such as inhibiting platelet aggregation, disrupting neurotransmission, and impairing vasodilation, with its acute exposure exhibiting more pronounced toxic effects. While previous studies have predominantly focused on high-concentration CO poisoning events, our findings indicate that chronic, low-dose exposure to CO at ambient concentrations remains a significant, non-negligible risk factor for stroke. NO₂ is also significantly associated with stroke incidence (*RR* = 1.057, 95% *CI*: 1.017–1.098), consistent with prior studies. Zhou et al. (8) similarly reported a strong association between short-term NO₂ exposure and emergency stroke visits in a Shanghai time-series study. Furthermore, Toubasi et al.’s (20) systematic review and meta-analysis explicitly identified NO₂ as one of the most significant risk factors for ischemic stroke onset. The underlying mechanism may involve inducing oxidative stress, disrupting vascular endothelial barrier integrity, and promoting atherosclerosis progression and thrombosis (26, 27). This mechanism remains effective even at low exposure concentrations, further highlighting the potential threat of gaseous pollutants to cerebrovascular health.

This study found that even in areas like Guiyang City, where pollution levels are lower than most major cities in China, the association between pollutants and stroke incidence remains significant, consistent with findings from other regions with high pollutant concentrations (16–19). For example, an analysis comparing PM_2.5_ and PM_10_ exposure in areas with high PM concentrations revealed that short-term exposure to environmental PM_2.5_ and PM_10_ is associated with increased mortality from cerebrovascular disease (28). This study also demonstrated that PM_2.5_ and PM_10_ were statistically significantly associated with stroke incidence among residents throughout the study period. Our findings are supported by extensive toxicological and epidemiological research. For instance, studies indicate that even low-level PM exposure negatively impacts vascular endothelial function, increases systemic inflammation and platelet activation, and reduces antioxidant enzyme activity (29, 30). These changes may promote blood coagulation and thrombosis, impair vascular function and blood flow, and lead to elevated blood pressure, ultimately potentially contributing to stroke occurrence. This finding fills a research gap regarding the health effects of air pollution at low contamination levels. It aligns with the conclusions of Kuźma et al. (31) in non-industrial areas of Poland, confirming that the harmful effects of air pollution on stroke are not confined to highly polluted regions. Even in relatively clean environments like Guiyang City, attention must be paid to its potential impact on public health. A review comprehensively cataloged experimental and epidemiological evidence, clearly demonstrating that PM_2.5_ significantly exacerbates stroke risk by inducing oxidative stress through the ERK/JAK/NFκB pathway, upregulating adhesion molecules, and promoting vascular endothelial inflammation (32). This may stem from the characteristics of air pollution, weather patterns, and economic factors. Therefore, it is reasonable to summarize its impacts based on geographic location.

The results from the dual-pollutant model suggest that when PM_2.5_ and NO_2_ are adjusted for each other, the estimated effects of these pollutants on stroke incidence in the general population are attenuated and no longer statistically significant. Although the VIF values were low (<5), this attenuation suggests that the effects of PM_2.5_ and NO_2_ on stroke are not entirely independent. Because they share emission sources (such as traffic combustion) and involve similar biological pathways related to oxidative stress and systemic inflammation, their effects may be partially confounded (33). The loss of statistical significance may also reflect reduced statistical power resulting from the inclusion of additional variables in the model, given the limited sample size and relatively low daily number of stroke cases. The effect of CO on stroke incidence in the general population showed a marked attenuation after adjusting for PM_2.5_ and NO_2_, with the RR decreasing by more than 20%, from a significant 1.346 to a non-significant 1.068. Given that CO had the lowest VIF value (1.66) across all tested models, this sharp decline strongly suggests that the significant association with CO observed in single-pollutant models likely serves merely as a proxy for a broader mixture of traffic-related pollutants (particularly PM_2.5_ and NO_2_) (34). This finding is consistent with our understanding of CO in ambient air, which is typically a marker of combustion efficiency and is co-emitted with particulate matter and nitrogen oxides from vehicle exhaust (35). Therefore, its independent toxic effects on cerebrovascular health are likely limited, and its effects observed in single-pollutant models should be interpreted with caution.

Identifying susceptible subgroups holds significant scientific and public health implications, as it suggests that targeted and personalized preventive measures for these potentially vulnerable populations may yield greater health benefits (31). This study conducted gender- and age-stratified subgroup analyses for six air pollutants (PM₂.₅, PM₁₀, SO₂, NO₂, CO, O₃), systematically elucidating susceptibility among different populations and its potential physiological mechanisms. Findings revealed that effect estimates for all six pollutants were consistently higher among women and older adults, suggesting these subgroups may be more susceptible. However, as formal interaction tests were not performed, these conclusions remain preliminary. Observed subgroup differences may arise from chance or confounding factors, necessitating larger-scale studies to confirm whether gender and age genuinely modify the impact of air pollution on stroke risk. Such disparities may also primarily stem from heterogeneity in physiological characteristics, baseline health status, and environmental exposure response mechanisms (32).

Regarding gender differences, the study findings indicate that women show stronger associations with short-term exposure across all six pollutants. Specifically, CO demonstrated a lag-0 *RR* as high as 1.548 (95% *CI*: 1.116–2.147), whereas men showed significant effects only at specific lag times for NO₂ and CO. A cohort study by Sun et al. (36) on postmenopausal women in the United States also found that women were more sensitive to stroke risks caused by air pollution, corroborating the conclusions of this study. This phenomenon may be related to the regulatory role of female estrogen levels, consistent with previous research. An animal model study indicated that, besides binding to hemoglobin and causing tissue hypoxia, the physiological effects of carbon monoxide (CO) at low concentrations (such as vasodilation and influencing neurotransmission) may exert unique impacts on the estrogen-regulated vascular system, thereby inducing more acute pathophysiological responses in females (25). Furthermore, physiological structural differences in the female respiratory and cardiovascular systems—such as smaller airway diameters and thinner vascular walls—may lead to heightened sensitivity to pollutant inhalation doses and toxicity responses (37), providing a plausible biological explanation for these gender disparities.

Additionally, the findings of this study indicate that individuals aged ≥65 years exhibit immediate (lag0) or cumulative delayed effects for the vast majority of pollutants, whereas those <65 years are only affected by short-term impacts of NO₂ and PM₁₀. The heightened sensitivity among older adults primarily stems from two factors: first, natural decline in vascular function, increased incidence of atherosclerosis, and reduced structural integrity and regulatory capacity of cerebral blood vessels, rendering them more vulnerable to pollutant-induced oxidative stress and inflammatory responses (11, 38); second, the prevalence of comorbidities such as hypertension and diabetes among older adults, which synergize with air pollution exposure to further elevate stroke risk (39, 40). In this study, the *RR* value for SO₂ exposure among individuals aged ≥65 years (1.130, 95% *CI*: 1.060–1.205) was significantly higher than that for the general population, consistent with findings by Chen et al. (41). This confirms the priority status of older adults in air pollution health risk management. Differences in susceptibility across genders and age groups may relate to their physiological characteristics and lifestyles. Older adults with weakened immune systems and underlying chronic conditions may exhibit heightened sensitivity to air pollution exposure.

In summary, this study analyzed the correlation between air pollutants and stroke incidence in Guiyang City from 2016 to 2021. The findings indicate that exposure to air pollutants (PM_2.5_, PM_10_, SO₂, NO₂, O₃, CO) all associated with increased risk of stroke among residents, with women and older adults being particularly susceptible. To address the potential health impacts of stroke and reduce the economic burden on families and society, it may be beneficial to implement air pollution control measures, allocate healthcare resources appropriately, and ensure high-risk populations take effective protective measures on days with high pollution levels. This study possesses several strengths. First, it represents the first investigation in a typical low-pollution, high-altitude karst topography city in Southwest China, filling a gap in research on the association between air pollution and stroke incidence under unique geographical and climatic conditions. Guiyang’s karst terrain restricts pollutant dispersion, while its plateau monsoon climate results in significantly different exposure patterns compared to plain cities. These findings provide scientific evidence for health impact assessments in similar regions. Second, while previous studies on air pollution and stroke predominantly used mortality as the outcome measure, few have focused on incidence with shorter time spans. This study employs population-based incidence data as the outcome indicator, which is more sensitive than mortality rates. The six-year longitudinal data further enhances the stability and reliability of the findings. Third, the analysis adopted a comprehensive approach. It not only examined the single-day and cumulative lag effects of six pollutants but also identified susceptible populations through gender and age stratification, providing detailed evidence for targeted prevention and control strategies.

Despite the significant public health implications of this study, several limitations exist. First, as this study employed a time-series analysis within an ecological framework, the subjects were based on populations rather than individuals, which may lead to ecological fallacy. Ambient monitoring data may not capture intra-urban variability in pollutant concentrations or individual-level exposure patterns influenced by time-activity budgets, indoor air pollution sources (e.g., cooking, heating, tobacco smoke), and proximity to traffic or industrial sources. Such non-differential exposure misclassification typically biases effect estimates towards the null, potentially leading to an underestimation of the true associations between air pollution and stroke risk. Second, the lack of stratified analysis by disease subtype (ischemic versus hemorrhagic stroke) prevents identification of potential differences in pollutant effects between these stroke types. The stroke incidence data were obtained from the national chronic disease surveillance system, where a substantial proportion of cases were recorded under the broad category of cerebrovascular disease (ICD-10 I64, ‘stroke, not specified as hemorrhage or infarction’) without mandatory subtype specification during the study period. This precluded reliable subtype classification without introducing misclassification bias. Additionally, the relatively low daily counts of hemorrhagic stroke would have resulted in unstable model estimates. The combination of ischemic and hemorrhagic stroke as a single outcome may have diluted the true effect estimates if air pollutants have differential effects on these subtypes. Previous studies suggest that ischemic stroke may be more strongly associated with air pollution through mechanisms involving thrombosis and atherosclerosis, while hemorrhagic stroke might show different or even null associations (22, 28). Further large-scale studies with more granular clinical data are needed to examine pollutant impacts on distinct stroke subtypes. Finally, although our generalized additive model controlled for temperature, humidity, long-term trends, day-of-week effects, and holiday effects, several potential confounders were not adjusted for. These include: (1) Influenza outbreaks: Due to the lack of daily city-level influenza surveillance data, we could not adjust for influenza epidemics, which are more common in winter and may trigger stroke. If influenza outbreaks coincide with high pollution days, the omission could lead to overestimation of pollution effects, though the smooth time trend in the model partially captures seasonal influenza patterns. (2) Socioeconomic factors: Individual-level variables such as income and education were unavailable in the aggregated data, which may introduce confounding bias of uncertain direction. (3) Population aging trends: This is a long-term trend that was effectively controlled by the smooth function of time and is unlikely to cause residual confounding. (4) Seasonal behavioral changes: Factors such as outdoor activity time and dietary patterns were not directly measured. Although meteorological variables and time trends partly reflect seasonal patterns, residual confounding may still exist, with direction depending on the specific behavior. (5) Indoor air exposure: Ambient monitoring data were used as a proxy for personal exposure, which does not account for indoor pollution sources (e.g., cooking, heating, tobacco smoke). This non-differential exposure misclassification likely biases effect estimates towards the null, potentially underestimating true associations. Future studies with more detailed individual-level data and indoor exposure assessmensuggestanted to validate our findings and suggesting that future studies incorporate spatial interpolation or satellite-based models to better capture intra-urban exposure contrasts.

The findings of this study originate from Guiyang, a low-pollution city with typical karst topography; however, their external validity requires us to interpret them with caution within specific contexts. Regarding other Chinese cities, our results are most applicable to areas with low pollutant concentrations, complex topography (such as basin or valley cities), and similar levels of socioeconomic development (42). For cities with higher pollutant concentrations, more complex sources, or flat topography, the strength of the association may differ. For low-pollution environments globally, this study provides new evidence from karst regions, supporting the growing body of evidence that the health effects of air pollution persist even at levels below regulatory standards (43, 44). Although pollution levels in Guiyang are relatively low, the effect sizes (e.g., a 10 μg/m^3^ increase in PM_2.5_ yielding an *RR* of 1.026) are largely consistent with findings from multiple low-pollution regions worldwide, suggesting that air pollution-induced stroke (through mechanisms such as oxidative stress and inflammation) may have biological universality across populations (45). However, the karst topography may lead to frequent temperature inversions and poor atmospheric dispersion conditions (13); therefore, caution is warranted when extrapolating these findings to plains or regions with markedly different meteorological conditions. Future multicenter studies encompassing diverse geographic environments, pollution levels (46), and climatic conditions should be conducted to further validate and quantify the generalizability of these findings.

## 5 Conclusion

The findings of this study indicate that in cities like Guiyang, characterized by low pollution levels but unique topography, short-term exposure to PM₂.₅, PM₁₀, SO₂, NO₂, O₃, and CO all associated with increased risk of stroke onset, with a delayed effect. Women and individuals aged 65 years and older appear to be more susceptible to these associations. It is recommended to strengthen air pollution control and health protection for high-risk groups, particularly by implementing targeted interventions during periods of elevated pollutant concentrations. This approach aims to reduce the burden of stroke and enhance public health standards.

## Data Availability

The data analyzed in this study is subject to the following licenses/restrictions: the datasets presented in this article are managed in strict accordance with the data security and confidentiality requirements imposed by the Guizhou Center for Disease Control and Prevention. Requests to access these datasets should be directed to gzcdc_gws@163.com.
