# Supplementary material for: Time-series analysis of the impact of short-term exposure to atmospheric pollutants on stroke risk among residents in Guiyang City, 2016–2021
Source: Front Public Health. 2026 Oct 16;14:1777772. doi: 10.3389/fpubh.2026.1777772 (PMC13719484; doi:10.3389/fpubh.2026.1777772)
Supplement: Supplementary file 1 [file Data_Sheet_1.docx]

**TABLE S1** AIC values

| time_df_year | temp_df | hum_df | AIC |
| --- | --- | --- | --- |
| 7 | 3 | 3 | 9612.98433 |
| 8 | 5 | 5 | 9614.58604 |
| 8 | 5 | 4 | 9615.24969 |
| 8 | 4 | 3 | 9616.88634 |
| 8 | 4 | 5 | 9618.24140 |
| 8 | 4 | 4 | 9618.95591 |
| 8 | 3 | 3 | 9636.81471 |
| 8 | 3 | 5 | 9637.75367 |
| 8 | 3 | 4 | 9638.47267 |
| 7 | 5 | 3 | 9645.81823 |
| 7 | 5 | 5 | 9647.33656 |
| 7 | 5 | 4 | 9647.99036 |
| 7 | 4 | 3 | 9651.59595 |
| 7 | 4 | 5 | 9652.85639 |
| 7 | 4 | 4 | 9653.52778 |
| 6 | 5 | 5 | 9668.38701 |
| 6 | 5 | 3 | 9668.49595 |
| 6 | 5 | 4 | 9670.06129 |
| 8 | 5 | 3 | 9670.36225 |
| 7 | 3 | 5 | 9671.27404 |
| 7 | 3 | 4 | 9671.83368 |
| 6 | 4 | 5 | 9675.34832 |
| 6 | 4 | 3 | 9675.92283 |
| 6 | 4 | 4 | 9677.10950 |
| 6 | 3 | 5 | 9697.67087 |
| 6 | 3 | 3 | 9698.93143 |
| 6 | 3 | 4 | 9699.43827 |

**TABLE S2 Dispersion parameter**

| Outcome | Pollutant | Dispersion | Deviance |
| --- | --- | --- | --- |
| Total | PM_2.5_ | 1.416 | 0.754 |
| Total | PM_10_ | 1.416 | 0.754 |
| Total | NO_2_ | 1.41 | 0.754 |
| Total | SO_2_ | 1.411 | 0.754 |
| Total | CO | 1.414 | 0.754 |
| Total | O_3_ | 1.417 | 0.753 |
| Male | PM_2.5_ | 1.225 | 0.669 |
| Male | PM_10_ | 1.225 | 0.669 |
| Male | NO_2_ | 1.223 | 0.669 |
| Male | SO_2_ | 1.224 | 0.669 |
| Male | CO | 1.225 | 0.669 |
| Male | O_3_ | 1.226 | 0.669 |
| Female | PM_2.5_ | 1.248 | 0.575 |
| Female | PM_10_ | 1.248 | 0.575 |
| Female | NO_2_ | 1.244 | 0.575 |
| Female | SO_2_ | 1.242 | 0.575 |
| Female | CO | 1.246 | 0.575 |
| Female | O_3_ | 1.246 | 0.575 |
| ＜65 years old | PM_2.5_ | 1.159 | 0.572 |
| ＜65 years old | PM_10_ | 1.158 | 0.572 |
| ＜65 years old | NO_2_ | 1.156 | 0.572 |
| ＜65 years old | SO_2_ | 1.161 | 0.571 |
| ＜65 years old | CO | 1.158 | 0.572 |
| ＜65 years old | O_3_ | 1.157 | 0.572 |
| ≥65 years old | PM_2.5_ | 1.266 | 0.671 |
| ≥65 years old | PM_10_ | 1.266 | 0.671 |
| ≥65 years old | NO_2_ | 1.263 | 0.672 |
| ≥65 years old | SO_2_ | 1.257 | 0.673 |
| ≥65 years old | CO | 1.264 | 0.671 |
| ≥65 years old | O_3_ | 1.267 | 0.671 |

**TABLE S3** Relative risks (95% *CI*) of stroke incidence associated with air pollutant exposure across different lag days (total population).

| **lag days** | *RR*(95%*CI*) | | | | | |
| --- | --- | --- | --- | --- | --- | --- |
|  | PM_2.5_ | PM_10_ | SO_2_ | NO_2_ | CO | O_3_ |
| **single-day lag** |  |  |  |  |  |  |
| Lag0 | ^b^1.026（1.005~1.048） | ^b^1.015（1.001~1.030） | ^b^1.075（1.016~1.137） | ^b^1.057（1.017~1.098） | ^b^1.346（1.072~1.691） | 1.009（0.997~1.022） |
| Lag1 | 1.014（0.994~1.034） | 1.009（0.996~1.021） | 1.039（0.985~1.097） | 1.015（0.977~1.053） | 1.058（0.842~1.330） | ^b^1.013（1.003~1.023） |
| Lag2 | 1.003（0.985~1.022） | 1.002（0.991~1.014） | 1.011（0.960~1.064） | 0.992（0.957~1.027） | 0.884（0.710~1.101） | 1.006（0.997~1.015） |
| Lag3 | 1.001（0.983~1.019） | 1.001（0.990~1.012） | 1.013（0.964~1.064） | 1.020（0.986~1.055） | 0.898（0.724~1.113） | 1.001（0.992~1.010） |
| Lag4 | 1.014（0.996~1.032） | 1.007（0.996~1.018） | 1.028（0.980~1.079） | ^b^1.044（1.010~1.079） | 1.065（0.862~1.316） | 1.000（0.992~1.009） |
| Lag5 | 0.998（0.981~1.017） | 0.998（0.987~1.009） | 1.003（0.955~1.052） | 1.013（0.980~1.048） | 0.946（0.765~1.169） | 1.000（0.991~1.009） |
| Lag6 | 0.999（0.981~1.017） | 0.998（0.988~1.009） | 0.975（0.928~1.024） | 0.992（0.958~1.026） | 0.978（0.791~1.209） | 1.003（0.995~1.012） |
| Lag7 | 1.002（0.984~1.020） | 0.999（0.989~1.010） | 0.986（0.940~1.035） | 0.999（0.966~1.034） | 0.928（0.750~1.147） | 0.997（0.989~1.006） |
| **Cumulative**  **lag** |  |  |  |  |  |  |
| Lag01 | ^b^1.027（1.004~1.051） | ^b^1.016（1.001~1.032） | ^b^1.078（1.013~1.147） | ^b^1.047（1.002~1.093） | 1.273（0.977~1.659） | ^b^1.015（1.002~1.028） |
| Lag02 | 1.025（0.999~1.051） | 1.015（0.999~1.031） | ^b^1.075（1.003~1.152） | 1.039（0.990~1.089） | 1.157（0.860~1.557） | ^b^1.014（1.000~1.028） |
| Lag03 | 1.024（0.997~1.051） | 1.014（0.997~1.031） | ^b^1.082（1.004~1.166） | ^b^1.056（1.004~1.111） | 1.130（0.816~1.564） | 1.012（0.998~1.027） |
| Lag04 | ^b^1.033（1.004~1.062） | ^b^1.019（1.001~1.037） | ^b^1.098（1.014~1.189） | ^b^1.076（1.019~1.135） | 1.240（0.871~1.765） | 1.012（0.998~1.027） |
| Lag05 | 1.026（0.996~1.057） | 1.014（0.995~1.033） | ^b^1.089（1.001~1.184） | ^b^1.064（1.006~1.127） | 1.167（0.800~1.700） | 1.011（0.997~1.026） |
| Lag06 | 1.026（0.995~1.059） | 1.014（0.995~1.034） | 1.073（0.981~1.173） | 1.056（0.995~1.121） | 1.191（0.799~1.774） | 1.013（0.998~1.028） |
| Lag07 | 1.029（0.995~1.063） | 1.014（0.994~1.035） | 1.083（0.985~1.191） | 1.061（0.996~1.131） | 1.142（0.751~1.738） | 1.010（0.995~1.026） |

Note: The unit of concentration is 10 μg/m³ for PM₂.₅, PM₁₀, NO₂, SO₂, and O₃, and 1 mg/m³ for CO. ^b^*P*< 0.05.

**TABLE S4** Results of multiple comparison correction (FDR, lag0, total population)

| Pollutant | Raw *P*-value | FDR-corrected P-value | FDR < 0.05 |
| --- | --- | --- | --- |
| PM_2.5_ | 0.0155 | 0.0232 | Yes |
| PM_10_ | 0.0304 | 0.0365 | Yes |
| SO_2_ | 0.0116 | 0.0232 | Yes |
| NO_2_ | 0.0046 | 0.0232 | Yes |
| CO | 0.0107 | 0.0232 | Yes |
| O_3_ | 0.1369 | 0.1369 | No |

Note: The Benjamini-Hochberg method was used to control the false discovery rate.

**TABLE S5** Maximum lag effects of ambient air pollutants on stroke incidence among residents of different sexes in Guiyang City, 2016–2021.

| Pollutant | male | | female | |
| --- | --- | --- | --- | --- |
|  | Maximum lag time | *RR*（95%*CI*） | Maximum lag time | *RR*（95%*CI*） |
| Maximum single-day lag effect | | | | |
| PM_2.5_ | Lag7 | 1.021（0.999~1.043） | Lag0 | *1.040（1.009~1.071） |
| PM_10_ | Lag7 | 1.012（0.999~1.012） | Lag0 | *1.023（1.003~1.043） |
| SO_2_ | Lag0 | 1.048（0.976~1.125） | Lag0 | *1.109（1.026~1.200） |
| NO_2_ | Lag4 | *1.056（1.015~1.100） | Lag0 | *1.067（1.009~1.127） |
| CO | Lag4 | *1.327（1.026~1.717） | Lag0 | *1.548（1.116~2.147） |
| O_3_ | Lag1 | 1.012（0.999~1.024） | Lag0 | *1.022（1.005~1.040） |
| Maximum cumulative lag effect | | | | |
| PM2.5 | Lag07 | 1.033（0.992~1.076） | Lag04 | *1.050（1.008~1.094） |
| PM10 | Lag07 | 1.019（0.994~1.044） | Lag01 | *1.024（1.002~1.064） |
| SO2 | Lag07 | 1.091（0.971~1.226） | Lag04 | *1.171（1.046~1.310） |
| NO2 | Lag07 | *1.090（1.009~1.178） | Lag03 | *1.076（1.000~1.158） |
| CO | Lag07 | 1.455（0.870~2.433） | Lag01 | 1.383（0.946~2.022） |
| O3 | Lag01 | 1.006（0.990~1.022） | Lag01 | *1.026（1.007~1.046） |

**Note:** * indicates a statistically significant result. Lag0 to Lag7 represent single-day lags, while Lag01 to Lag07 represent cumulative lags.

**TABLE S6** Maximum lag effects of ambient air pollutants on stroke incidence among residents of different age groups in Guiyang City, 2016-2021.

| Pollutant | ≥65 years old | | ＜65 years old | |
| --- | --- | --- | --- | --- |
|  | Maximum lag time | RR（95%CI） | Maximum lag time | RR（95%CI） |
| Maximum single-day lag effect | | | | |
| PM_2.5_ | Lag0 | 1.029（1.003~1.055）^*^ | Lag4 | 1.024（0.997~1.051） |
| PM_10_ | Lag0 | 1.016（1.000~1.033）^*^ | Lag4 | 1.016（0.999~1.032） |
| SO_2_ | Lag0 | 1.130（1.060~1.205）^*^ | Lag4 | 1.061（0.989~1.140） |
| NO_2_ | Lag0 | 1.059（1.012~1.108）^*^ | Lag4 | 1.070（1.018~1.124）^*^ |
| CO | Lag0 | 1.312（1.001~1.720）^*^ | Lag0 | 1.401（0.994~1.974） |
| O_3_ | Lag1 | 1.013（1.001~1.025）^*^ | Lag4 | 1.061（0.989~1.140） |
| Maximum cumulative lag effect | | | | |
| PM_2.5_ | Lag01 | 1.025（0.997~1.053） | Lag07 | 1.050（0.999~1.103） |
| PM_10_ | Lag01 | 1.014（0.997~1.032） | Lag04 | 1.027（1.000~1.055）^*^ |
| SO_2_ | Lag01 | 1.129（1.051~1.213） | Lag04 | 1.032（0.913~1.167） |
| NO_2_ | Lag01 | 1.049（0.997~1.104） | Lag04 | 1.013（1.018~1.196）^*^ |
| CO | Lag01 | 1.219（0.890~1.669） | Lag01 | 1.363（0.890~1.669） |
| O_3_ | Lag01 | 1.013（0.997~1.028） | Lag01 | 1.018（0.998~1.038） |

**Note:** * indicates a statistically significant result. Lag0 to Lag7 represent single-day lags, while Lag01 to Lag07 represent cumulative lags.

**TTABLE S7** Results of period-specific sensitivity analysis for COVID-19 (total population, lag 0)

| Period | Pollutant | *RR* (95% *CI*) | *P*-value |
| --- | --- | --- | --- |
|  | PM₂.₅ | 1.034 (1.006–1.064) | 0.019 |
|  | PM₁₀ | 1.029 (1.008–1.050) | 0.006 |
| **Pre-pandemic (2016–2019)** | NO₂ | 1.073 (1.018–1.131) | 0.009 |
|  | SO₂ | 1.098 (1.020–1.183) | 0.013 |
|  | CO | 1.232 (0.898–1.689) | 0.196 |
|  | O₃ | 1.006 (0.985–1.027) | 0.585 |
|  | PM₂.₅ | 1.034 (1.001–1.067) | 0.045 |
|  | PM₁₀ | 1.012 (0.992–1.032) | 0.251 |
| **Pandemic (2020–2021)** | NO₂ | 1.064 (1.005–1.128) | 0.034 |
|  | SO₂ | 1.089 (0.997–1.189) | 0.059 |
|  | CO | 1.364 (0.973–1.912) | 0.072 |
|  | O₃ | 1.014 (0.998–1.030) | 0.091 |
|  | PM₂.₅ | 1.026 (1.005–1.048) | 0.015 |
|  | PM₁₀ | 1.015 (1.001–1.030) | 0.030 |
| **Full period (2016–2021)** | NO₂ | 1.057 (1.017–1.098) | 0.005 |
|  | SO₂ | 1.075 (1.016–1.137) | 0.012 |
|  | CO | 1.346 (1.072–1.691) | 0.011 |
|  | O₃ | 1.009 (0.997–1.022) | 0.137 |

Supplementary Figure 1. autocorrelation function (PACF) plots

Supplementary Figure 2. Residuals vs. fitted values

Supplementary Figure 3. Residuals over time

Supplementary Figure 4. Partial residual plot for PM₂.₅

Supplementary Figure 5. Time series plot of stroke incidence in Guiyang City, 2016–2021

Supplementary Figure 6. Time series chart of atmospheric pollutants concentrations in Guiyang City, 2016–2021

Supplementary Figure 7. Time series chart of meteorological factors in Guiyang City, 2016–2021

Supplementary Figure 8. Heatmap of Spearman Correlation Coefficients between Air Pollutants and Meteorological Factors
